# Cardiomyocyte IL-1R2 protects heart from ischemia/reperfusion injury by attenuating IL-17RA-mediated cardiomyocyte apoptosis

**DOI:** 10.1038/s41419-022-04533-1

**Published:** 2022-01-27

**Authors:** Jun Lin, Qinfeng Li, Tingting Jin, Jiacheng Wang, Yingchao Gong, Qingbo Lv, Meihui Wang, Jiawen Chen, Min Shang, Yanbo Zhao, Guosheng Fu

**Affiliations:** 1 Department of Cardiology, Sir Run Run Shaw Hospital, School of Medicine, Zhejiang University, Hangzhou, China; 2 Key Laboratory of Cardiovascular Intervention and Regenerative Medicine of Zhejiang Province, Hangzhou, China

**Keywords:** Apoptosis, Myocardial infarction

## Abstract

Myocardial ischemia reperfusion (I/R) injury is a complex process with intense inflammatory response and cardiomyocyte apoptosis. As a decoy receptor of IL-1β, Interleukin-1 receptor type 2 (IL-1R2) inhibits IL-1β signaling. However, its role in I/R injury remains unknown. Here we found that the serum levels of IL-1R2 were significantly increased in patients with acute myocardial infarction (AMI) following interventional therapy. Similarly, after myocardial I/R surgery, IL-1R2 expression was significantly increased in heart of wild-type mice. In addition, IL-1R2-deficient mice heart showed enlarged infarct size, increased cardiomyocyte apoptosis together with reduced cardiac systolic function. Following exposure to hypoxia and reoxygenation (H/R), neonatal rat ventricular myocytes (NRVM) significantly increased IL-1R2 expression relying on NF-κB activation. Consistently, IL-1R2-deficient mice increased immune cells infiltrating into heart after surgery, which was relevant with cardiac damage. Additionally, IL-1R2 overexpression in cardiomyocyte protected cardiomyocyte against apoptosis through reducing the IL-17RA expression both in vivo and in vitro. Our results indicate that IL-1R2 protects cardiomyocytes from apoptosis, which provides a therapeutic approach to turn down myocardial I/R injury.

## Introduction

The high mortality rate of myocardial infarction has become an important health challenge, particularly taking into consideration that the rapid restoration of coronary flow even leads to salvage of remaining cardiomyocytes after a heart attack [1]. Unfortunately, reperfusion therapy unpredictably leads to sterile inflammation and cell death in the myocardium, causing tremendous cardiac injury [2–4]. Recently, emerging evidence has indicated that interleukin-17A (IL-17A) induces cardiomyocyte apoptosis by its ubiquitous receptor, IL-17RA, during myocardial I/R injury [5]. Although IL-17A is not expressed in cardiomyocytes, cardiomyocyte apoptosis is caused by macrophages and neutrophils released IL-17A via IL-17RA during I/R injury.

The Janus kinase (JAK) and signal transducer and activator of transcription (STATs) pathway plays critical roles in orchestrating immunity and cell death [6, 7]. Among the transcription factors in the STAT family, STAT1 and STAT3 have been reported to participate in the myocyte apoptosis during myocardial I/R injury. Hearts of cardiac-specific STAT3 knockout mice have larger infarct regions and more apoptotic cardiomyocytes than control mice [8, 9]. However, STAT1 knockdown mice show reduced myocyte death and decreased mortality [10]. Further research showed that STAT1 enhanced the transcription of pro-apoptotic mediators, such as Bax, Fas, and caspase-1 [11, 12].

Recent studies have reported that IL-1β blockade improved systolic function during myocardial infarction, suggesting that anti–IL-1β antibodies can be used to protect cardiomyocytes against myocardial I/R injury [13, 14]. IL-1R2, which lacks an intracellular Toll/interleukin-1 receptor (TIR) domain, is considered as a decoy receptor that prevents the intracellular IL-1β signaling cascade [15]. In addition to functioning as a membrane-bound receptor, IL-1R2 can be released into the circulatory system. The released isoform is known as soluble IL-1R2 (sIL-1R2) [16, 17]. As an interleukin receptor, IL-1R2 is usually expressed in leukocytes [18, 19]. Recent studies have reported that IL-1R2 was expressed in breast and colon cancer cells [20, 21]. Here, we hypothesize that cardiomyocyte expression of IL-1R2 is induced during the pathophysiology of myocardial I/R injury. To evaluate the expression of IL-1R2 upon myocardial I/R injury, plasma from AMI patients after interventional therapy and healthy controls, heart tissues, and blood samples from mice subjected to myocardial I/R injury were collected to measure the sIL-1R2 level. In addition, IL-1R2 knockout mice and IL-1R2-overexpressed AAV9 were used to explore the function and mechanisms of IL-1R2 in myocardial I/R injury.

## Results

### I/R injury induces IL-1R2 expression in heart and blood plasma

**Fig. 1:**
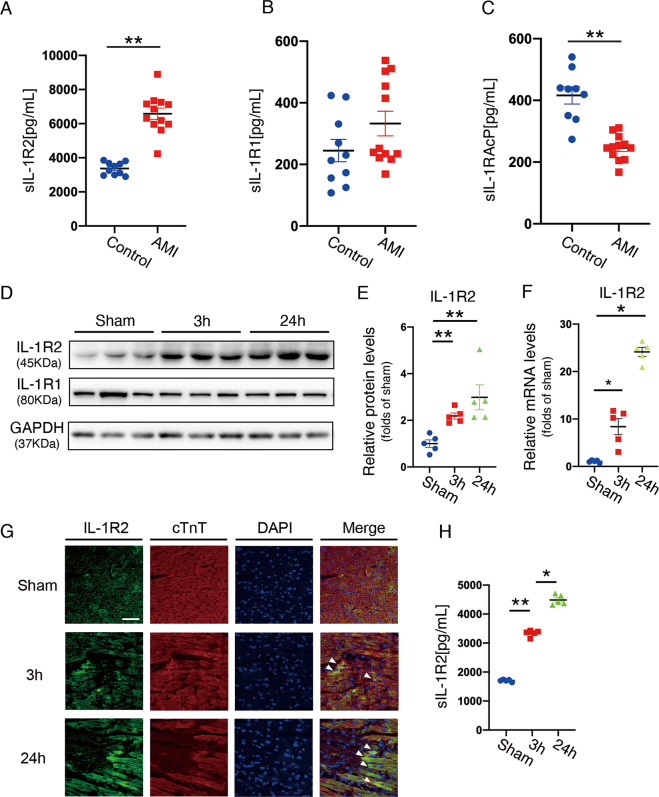
Plasma sIL-1R2 was elevated in acute myocardial infarction (AMI) patients undergoing interventional treatment. Blood plasma was collected from healthy donors (*n* = 10) and AMI patients after 24 h of interventional therapy (*n* = 12). **A–C** sIL-1R1, sIL-1R2, and sIL-1RAcP proteins levels were assessed by ELISA. **D–F** Immunoblots and quantification of IL-1R1 and IL-1R2 expression in the heart from mice after 3 h and 24 h of I/R surgery. **G** Representative images of IL-1R2 and cTnT staining on the heart tissue section from mice with sham surgery, 45 min of ischemia and 3 h of reperfusion surgery, 45 min of ischemia, and 24 h of reperfusion surgery (white arrows indicate the increased expression of IL-1R2 in cardiomyocyte around the ischemic area). **H** sIL-1R2 protein levels in mice with sham surgery, 3 h and 24 h of reperfusion in I/R surgery (*n* = 5). Scale bar: 100 µm. *P < 0.05 and **P < 0.01. Two-tailed unpaired Student’s *t*-test for two groups or one-way ANOVA for three groups or more.

To evaluate the expression of IL-1R2 upon myocardial I/R injury, we collected the plasma from 12 AMI patients under interventional therapy and 10 healthy donors as controls. Patient characteristics at baseline are shown in Supplemental Table 1. ELISA assay results revealed that sIL-1R2 levels dramatically increased in the peripheral blood of AMI patients under interventional therapy when compared to those in the controls (Fig. 1A). Whereas the level of sIL-1R1 was comparable in both groups (Fig. 1B). The level of soluble interleukin-1 receptor accessory protein (sIL-1RAcP), which is a co-receptor of interleukin-1 receptor for signal transduction, was significantly reduced in AMI patients under interventional therapy compared to healthy controls (Fig. 1C).

To further investigate whether the induction of sIL-1R2 upon myocardial I/R injury was derived from the heart, we subjected 8–10 weeks old mice to myocardial ischemia for 45 min followed by reperfusion for 3 and 24 h (Supplemental Fig. 1A). As expected, western blotting and quantitative reverse transcription-PCR (qRT-PCR) showed that the expression of *IL-1R2* was significantly increased after 3 and 24 h of reperfusion, whereas *IL-1R1* expression showed no significant change (Fig. 1D–F). Consistently, cardiomyocytes in the infarct area also increased IL-1R2 expression in mice subjected to myocardial I/R surgery by immunostaining (Fig. 1G). In addition, mice with 45 min of ischemia followed by 3 and 24 h of reperfusion increased sIL-1R2 level in blood compared with the sham group by ELISA assay (Fig. 1H). Taken together, these results indicate that I/R triggers IL-1R2 expression in the heart and sIL-1R2 release from injured cardiac myocytes.

### H/R stimulation significantly induced IL-1R2 expression in NRVM by NF-κB signaling

**Fig. 2:**
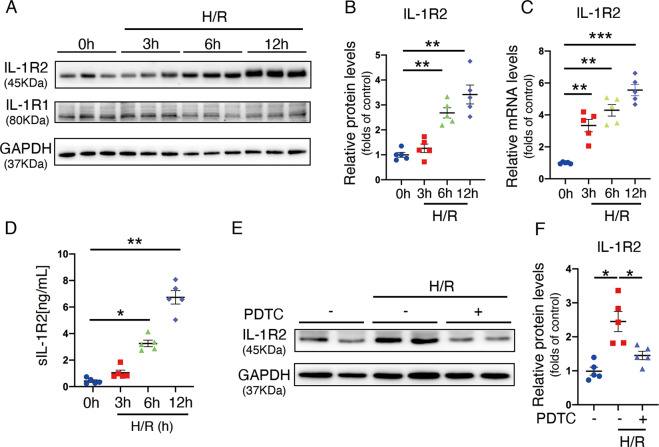
H/R treatment induces IL-1R2 expression in NRVM via NF-κB signaling. **A, B** Representative western blots of IL-1R2 and IL-1R1 (**A**) and quantification of IL-1R2 (**B**) in NRVM subjected to hypoxia for 3 h followed by reoxygenation for 3, 6, 12 h treatment (*n* = 5 per group). **C** qRT-PCR for IL-1R2 in NRVM with hypoxia for 3 h followed by reoxygenation for 3, 6, and 12 h treatment (*n* = 5 per group). **D** Quantification of ELISA assay for cell-culture medium collected from NRVM with hypoxia for 3 h followed by reoxygenation for 3, 6, and 12 h treatment (*n* = 5 per group). **E, F** Representative western blots and quantification of IL-1R2 in NRVM with 3 h of hypoxia, followed by reoxygenation of 12 h with or without PDTC treatment (*n* = 5 per group). *P < 0.05, **P < 0.01 and ***P < 0.001. Two-tailed unpaired Student’s *t*-test for two groups or one-way ANOVA for three groups or more.

To mimic I/R condition in vitro, NRVM was incubated in a hypoxia chamber with 94.8% N_2_/5% CO_2_/0.02% O_2_ in Esumi ischemic buffer for 3 h, followed by restoring in normal culture medium for 3–12 h as previously described [22] (Supplemental Fig. 1B). LDH assay confirmed that the cell death was time-dependent upregulated in the reoxygenation period (Supplemental Fig. 2A). Consistently, *IL-1R2* was increased in mRNA level from 3 to 12 h during culture medium restoration by qRT-PCR. The protein level was not increased during the first 3 h but significantly increased after 6 h until 12 h. However, *IL-1R1* expression in NRVM was not significantly changed neither in mRNA (data not shown) nor in protein levels upon stimulated I/R treatment (Fig. 2A–C). Furthermore, ELISA assay on cell-culture medium showed the level of sIL-1R2 was increased during the reoxygenation period, suggesting that cardiomyocyte increased sIL-1R2 release upon myocardial I/R injury from (Fig. 2D). To determine the mechanism of IL-1R2 induction, NRVM pre-treated with pyrrolidine dithiocarbamate (PDTC) to inhibit NF-κB phosphorylation was exposed to H/R stimulation. Phosphorylation of NF-κB induced by H/R was significantly reduced by PDTC treatment (Supplemental Fig. 2B, C). Compared with the control group, PDTC pre-treated NRVMs markedly reduced IL-1R2 expression upon H/R treatment (Fig. 2E, F). Collectively, these results show that the H/R treatment induces IL-1R2 expression and sIL-1R2 release by cardiomyocytes via NF-κB activation.

### IL-1R2 deficiency leads to cell death and cardiac function impairment via phosphorylation of STAT1

**Fig. 3:**
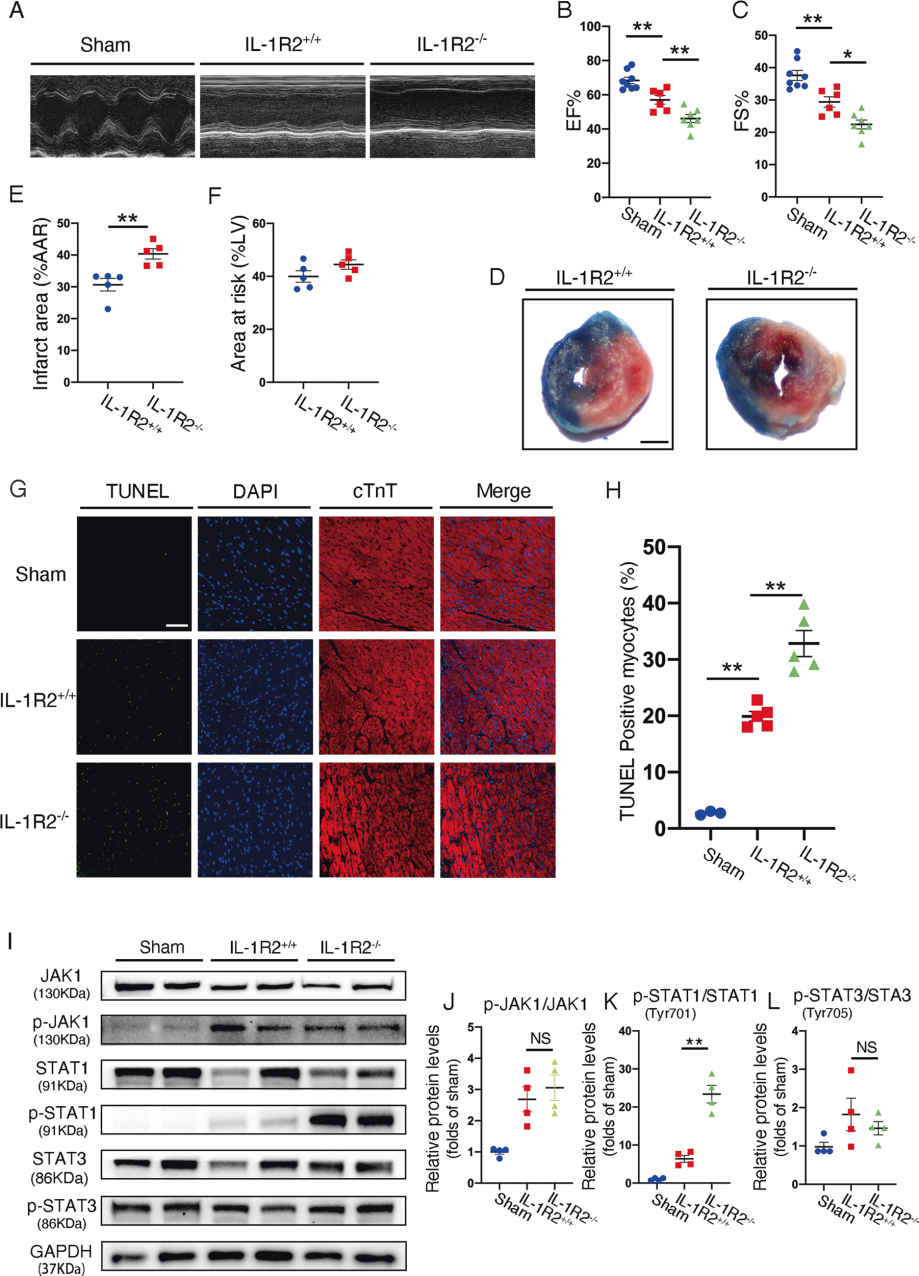
IL-1R2 knockout mice exacerbated cardiac damage in myocardial I/R injury. **A–C** Representative echocardiographic images and quantification for Sham (*n* = 8), IL-1R2+/+(*n* = 6) and IL-1R2−/− (*n* = 7) mice subjected to myocardial I/R surgery. **D–F** Representative images of 2,3,5-triphenyltetrazolium chloride staining in mice subjected to myocardial I/R surgery and quantification of the relative ratios of infarct area to area at risk (border zone) as well as the relative ratios of the area at risk to left ventricle area in IL-1R2+/+ (*n* = 5) and IL-1R2−/− (*n* = 5) mice. Scale bar: 1 mm. **G, H** Representative images and quantitation of TUNEL staining on the heart sections from the Sham (*n* = 3), IL-1R2+/+ (*n* = 5), and IL-1R2−/− (*n* = 5) mice. Scale bar: 100 µm. (**I–L**) Representative western blots and quantification of JAK1, p-JAK1, STAT1, p-STAT1, STAT3, and p-STAT3 in the heart from the Sham, IL-1R2+/+, and IL-1R2−/− mice subjected to myocardial I/R surgery. Scale bar: 100 µm. *P < 0.05 and **P < 0.01. Two-tailed unpaired Student’s *t*-test for two groups or one-way ANOVA for three groups or more.

We interbred IL-1R2^+/−^ heterozygous mice to generate global IL-1R2 knockout mice (IL-1R2^−/−^) and control littermates (IL-1R2^+/+^). Genotyping strategy was shown in supplemental information (Supplemental Fig. 2D). The deficiency of IL-1R2 in heart was confirmed by agarose gel electrophoresis and qRT-PCR (Supplemental Fig. 2E–G). IL-1R2^−/−^ mice and control littermates were administered with ischemia for 45 min followed by reperfusion for 24 h (Supplemental Fig. 1C). Echocardiographs demonstrated that ejection fractions (EF) and fractional shortening (FS) were significantly reduced in IL-1R2^−/−^ mice compared with those of littermate controls (Fig. 3A–C). TTC staining revealed that infarct areas were significantly increased in the hearts from IL-1R2^−/−^ mice compared with their control littermates upon myocardial I/R injury. The areas at risk were comparable in both genotypes (Fig. 3D–F). Additionally, TUNEL-positive cardiomyocytes were increased in IL-1R2^−/−^ mice compared with control littermates (Fig. 3G, H). JAK/STAT pathway has been reported to play a critical role in regulating cardiac myocyte apoptosis after myocardial I/R injury [8, 23]. Heart tissues, which were collected from mice with 45 min of ischemia followed by 3 h of reperfusion, expressed comparable level of phosphorylated JAK1 (p-JAK1) and tyrosine-phosphorylated STAT3 (p-STAT3) in both genotypes. However, the Tyr701 phosphorylation of STAT1 was markedly increased in IL-1R2^−/−^ mice compared with littermate controls (Fig. 3I–L). Taken together, these data indicate that IL-1R2 protects cardiomyocytes from I/R injury by suppressing STAT1 phosphorylation.

### IL-1R2^−/−^ mice enhanced monocytes infiltration and cytokines expression in the heart after I/R injury

**Fig. 4:**
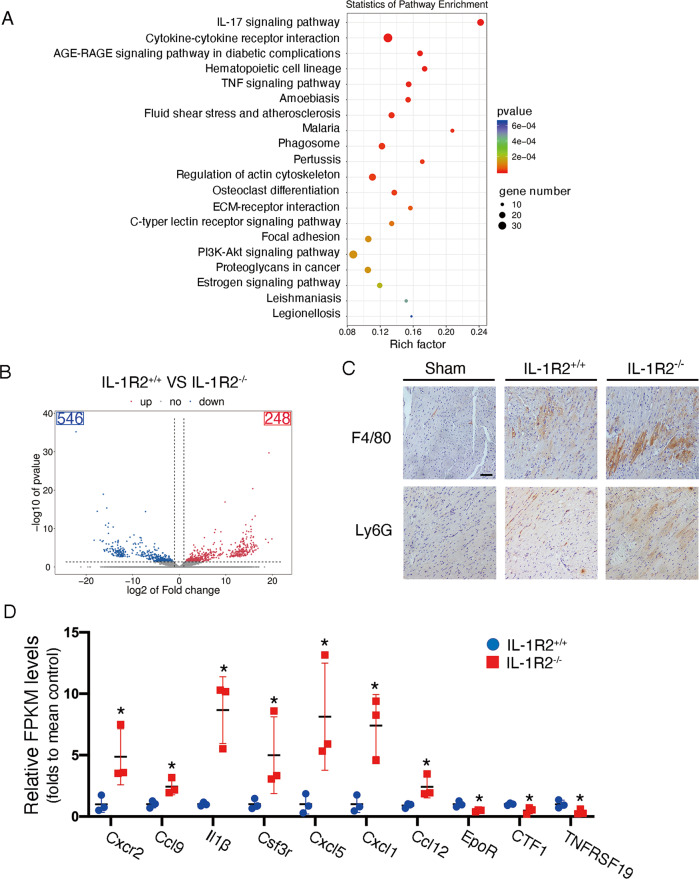
Knockout of IL-1R2 induces cytokines expression in the heart after I/R injury. **A** A bubble chart showing KEEG pathways enrichment in IL-1R2+/+ and IL-1R2−/− mice. **B** The volcano diagram displays the differentially expressed protein in IL-1R2+/+ and IL-1R2−/− mice. **C** Representative images of F4/80+ or Ly6G + areas in the heart through immunochemical staining of heart from Sham, IL-1R2+/+, and IL-1R2−/− mice subjected to myocardial I/R surgery. **D** Relative FPKM levels of chemokine genes related to cytokine-cytokine receptor interaction are shown (*n* = 3). The differentially expressed mRNAs and genes were selected with log2 (fold change) > 1 or log2 (fold change) < −1 and with statistical significance (*p*-value < 0.05).

Afterward, RNA sequencing analysis was performed on left ventricle samples from both IL-1R2^−/−^ mice and littermate controls subjected to 45 min of ischemia followed by 3 h of reperfusion. Differentially expressed genes between IL-1R2^-/-^ mice (*n* = 3) and littermate controls (*n* = 3) were analyzed by hierarchical clustering (Fig. 4A, supplemental Fig. 3A). Next, KEGG pathway analyses showed that cytokine-cytokine receptor interaction and IL-17 signaling were mediated by IL-1R2 upon myocardial I/R injury (Fig. 4A). By analyzing the differentially expressed genes between two genotypes, we identified 546 mRNAs were upregulated and 248 mRNAs were downregulated in IL-1R2^−/−^ mice compared with littermate controls (Fig. 4B). Additionally, we observed that the expression of F4/80 and Ly6G were significantly increased in the border zones of ischemic areas in IL-1R2^−/−^ mice compared with littermate controls after I/R injury (Fig. 4C). Consistently, pro-inflammation cytokines and their receptors were significantly increased in IL-1R2^−/−^ mice. Components associated with cardiac survival and proliferation, such as Erythropoietin receptor (EpoR), Cardiotrophin-1 (CTF1) and Tumor necrosis factor receptor superfamily, member 19 (TNFRSF19), were significantly decreased in IL-1R2^−/−^ mice compared with control mice (Fig. 4D). Moreover, the RAGE axis and TNF pathway, which were proved to participate in inflammatory response and cardiomyocyte apoptosis in I/R injury, also altered in IL-1R2 knockout mice upon I/R injury. (Supplemental Fig. 3B, C). Taken together, these data indicate that IL-1R2 downregulates inflammatory response and cell apoptosis to protect the cardiomyocyte from I/R injury.

### Cardiomyocyte IL-1R2 protects heart against apoptosis via downregulating IL-17RA expression upon myocardial I/R injury

**Fig. 5:**
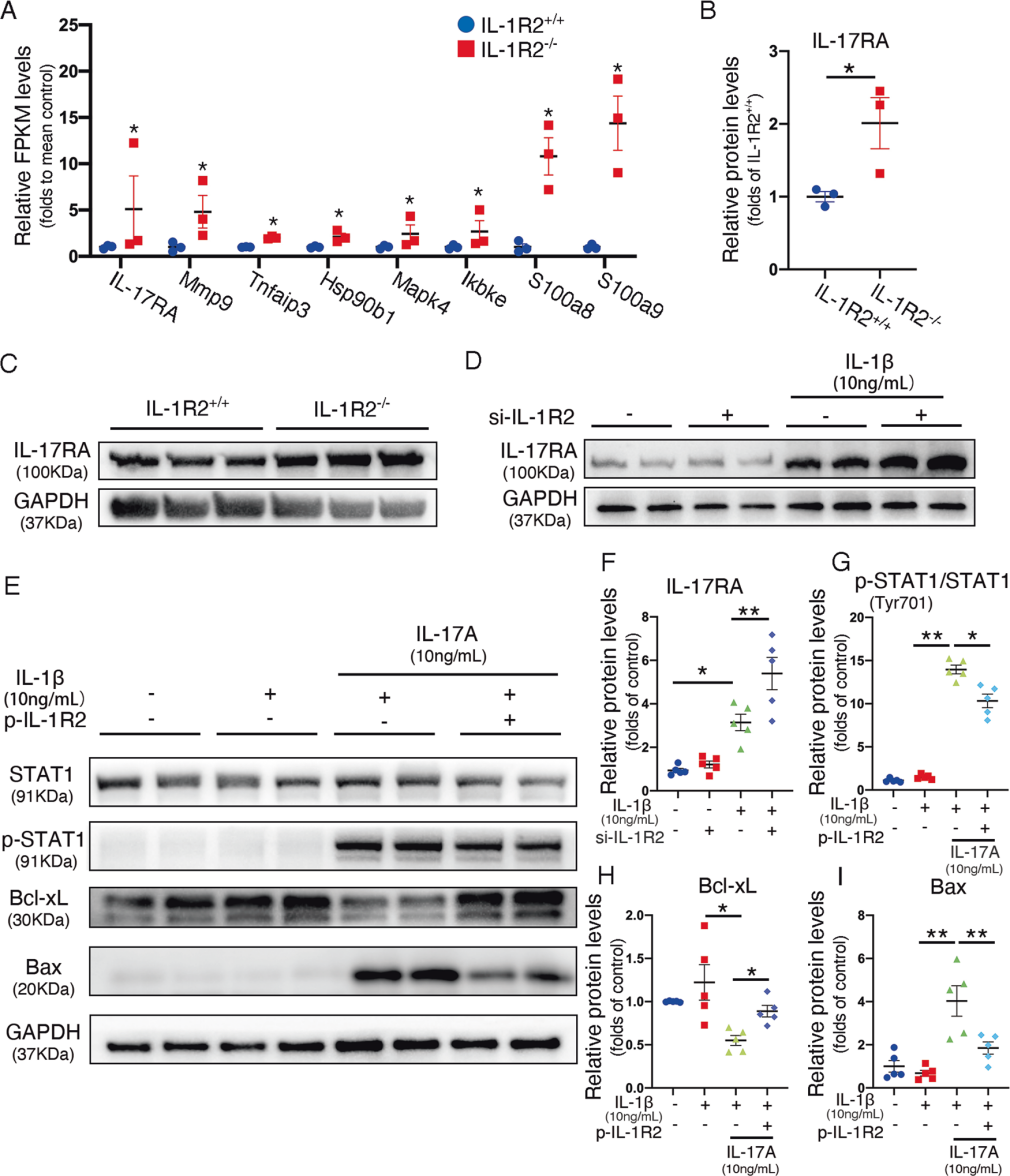
IL-1R2 protects heart against IL-17A-induced cardiomyocytes apoptosis through suppressing IL-17RA expression. **A** Relative FPKM levels of chemokine genes related to IL-17A signaling are shown (*n* = 3). The differentially expressed mRNAs and genes were selected with log2 (fold change) > 1 or log2 (fold change) < −1 and with statistical significance (*p*-value < 0.05). **B, C** Representative western blots and quantification of IL-17RA expression in the heart from IL-1R2+/+ and IL-1R2−/− mice subjected to myocardial I/R injury (*n* = 3 per group). **D, F** Representative western blots and quantification of IL-17RA expression in NRVMs transfected with siRNA to block IL-1R2 expression, followed by IL-1β treatment (*n* = 3 per group). **E, G–I** Representative western blots and quantification of STAT-1, p-STAT-1, Bcl-xL, and Bax expression in NRVMs with or without a plasmid transfection for overexpression of IL-1R2 followed by IL-1β and IL-17A treatment (*n* = 5 per group). *P < 0.05 and **P < 0.01. Two-tailed unpaired Student’s *t*-test for two groups or one-way ANOVA for three groups or more.

IL-1R2 knockout mice strongly induced IL-17 signaling and increased IL-17RA expression upon I/R injury (Fig. 5A–C). To determine the mechanism of IL-17RA induction we treated NRVMs with IL-1β in vitro. NRVMs increased IL-17RA expression during IL-1β treatment (Supplemental Fig. 4A), which was inhibited by P38 MAPK inhibitor (SB203580) but not JNK inhibitor (SP600125) (Supplemental Fig. 4B). These data indicate that IL-1β induces IL-17RA expression in cardiomyocyte via P38 MAPK pathway.

To further verify this observation, we used small interference RNA for IL-1R2 (si-IL-1R2) to knock down IL-1R2 (Supplemental Fig. 4C, Fig. 5H). IL-1R2 knockdown by siRNA transfection in LDH assay confirmed that IL-1R2 expression did not affect the cell necrosis caused by H/R (Supplemental Fig. 5I). IL-1β induced IL-17RA expression was further enhanced in IL-1R2 knockdown NRVM (Fig. 5D, F). IL-1R2 overexpressed NRVM was obtained by plasmid transfection and confirmed by western-blot assay (Supplemental Fig. 4D). Interestingly, sIL-1R2 level in the cell-culture medium was independent on IL-1R2 overexpression in NRVM (Supplemental Fig. 4E), which suggested that accumulated sIL-1R2 was not released by cardiomyocyte under normal conditions. IL-1β treatment did not change p-STAT1, Bcl-xL, or Bax expression in NRVM. However, combined treatment with IL-17A significantly increased p-STAT1 and Bax expression in NRVM, and reduced the Bcl-xL expression. Moreover, IL-1R2 overexpression in NRVM significantly reversed the upregulated expression of p-STAT1, Bax and downregulated expression of Bcl-xL induced by IL-17A and IL-1β treatment (Fig. 5E, G–I). Next, we performed flow cytometry to check cell apoptosis and found that IL-1R2 overexpressed NRVM reduced cell death upon IL-17A and IL-1β stimulation (Supplemental Fig. 3D, E). Similarly, TUNEL staining showed that IL-1R2 overexpression significantly decreased TUNEL-positive NRVM upon IL-17A and IL-1β treatment (Supplemental Fig. 4F, G). Taken together, these data indicate that IL-17RA induced cardiomyocyte apoptosis augmented by IL-1β was abrogated by IL-1R2 overexpression in cardiomyocyte.

### IL-1R2 overexpression in cardiomyocyte protects heart from myocardial I/R-induced apoptosis

**Fig. 6:**
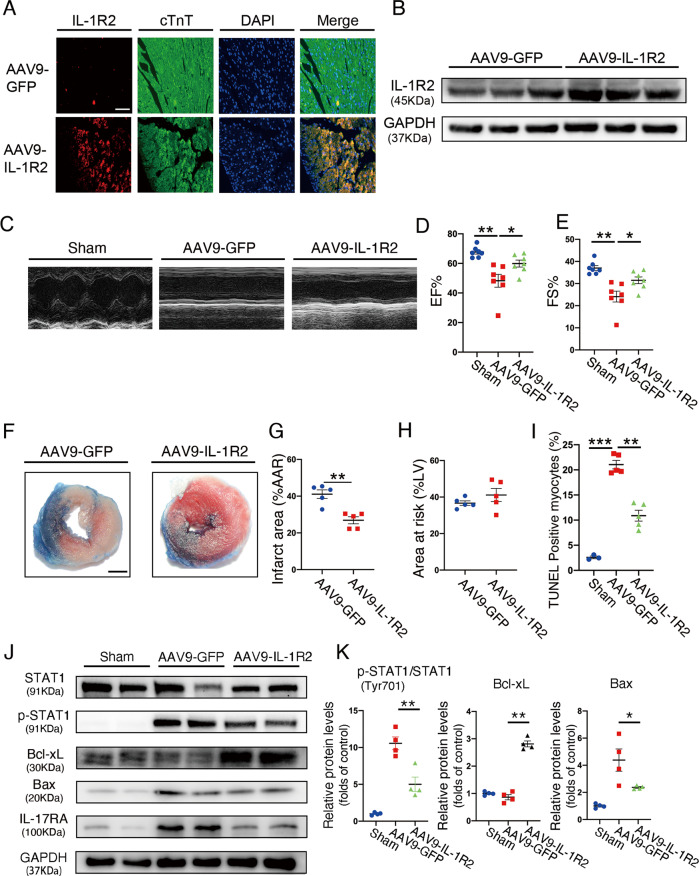
Overexpression of IL-1R2 in cardiomyocyte protects heart from I/R injury. **A, B** Representative images and western blots for confirming the expression of IL-1R2 in cardiomyocytes with AAV9-GFP and AAV9-IL-1R2 injection. **C–E** Representative echocardiographic images and quantification of sham mice (*n* = 7), GFP (*n* = 7) and IL-1R2-overexpression mice (*n* = 7) subjected to myocardial I/R surgery. **F–H** Representative images of 2,3,5-triphenyltetrazolium chloride staining on mice subjected to myocardial I/R surgery and quantification of the relative ratios of infarct area to area at risk (border zone) as well as the relative ratios of the area at risk to left ventricle area in the GFP (*n* = 5) and IL-1R2–overexpressing (*n* = 5) groups; Scale bar: 1 mm. **I** Quantitation of TUNEL staining on the heart sections from the Sham (*n* = 3), GFP (*n* = 5), and IL-1R2–overexpressing (*n* = 5) mice subjected to myocardial I/R surgery. Scale bar: 100 µm. **J, K** Representative western blots and quantification of Stat1, p-Stat1, Bcl-xL, Bax, and IL-17RA expression in the hearts from Sham, GFP, and IL-1R2–overexpressing mice subjected to myocardial I/R injury. Scale bar: 100 µm. *P < 0.05 and **P < 0.01. Two-tailed unpaired Student’s *t*-test for two groups or one-way ANOVA for three groups or more.

Furthermore, AAV9 carrying IL-1R2 under the cTnT promoter was administered to (5–7)-week-old male mice through myocardial injection to overexpress IL-1R2 in cardiomyocytes. Mice injected with AAV-GFP were used as negative controls (GFP) (Supplemental Fig. 1D). IL-1R2 was upregulated in the cardiomyocytes after 3 weeks of injection (Fig. 6A, B and supplemental Fig. 5G). The circulating level of sIL-1R2 did not change in IL-1R2-overexpressed mice (Supplemental Fig. 5B). EF of IL-1R2–overexpressed mice and GFP mice after 24 h of reperfusion were 56.02 ± 3.367 and 42.84 ± 2.957, respectively. Additionally, FS was significantly improved in IL-1R2–overexpressed mice compared with GFP mice (29 ± 2.226 vs 20.72 ± 1.712) (Fig. 6C–E). TTC staining revealed significantly smaller infarct areas in the IL-1R2-overexpressed hearts compared with controls, even though the areas at risk were comparable in both conditions (Fig. 6F–H). Similarly, IL-1R2-overexpressed mice reduced TUNEL-positive cardiomyocytes in myocardial area at risk (AAR) (Fig. 6I and Supplemental Fig. 5A). Besides, IL-1R2-overexpressed mice reduced the expressions of p-STAT1, IL-17RA, Bax, and in heart from mice with 45 min of ischemia followed by 3 h of reperfusion (Fig. 6J, K). These results further confirmed that IL-1R2 overexpression has an anti-apoptotic effect on cardiomyocytes upon myocardial I/R injury in vivo. Moreover, we evaluated the reactive oxygen species (ROS) levels of nitrotyrosine, which is related to cell damage and inflammation [24, 25]. Immunofluorescent and western-blot assays showed that myocardial I/R injury significantly increased nitrotyrosine level, which was reduced by IL-1R2 overexpression (Supplemental Fig. 5C–F). In aggregate, these data indicate that IL-1R2 overexpression in cardiomyocyte protects heart from I/R injury by turning down cell apoptosis.

## Discussion

Myocardial I/R injury is a common disease in patients undergoing interventional therapy. Although many studies have suggested that anti-inflammatory strategies protect cardiomyocytes from myocardial I/R injury [26, 27], very little is known about the underlying mechanisms. Here, we found that myocardial I/R strongly induces IL-1R2 expression in cardiomyocytes upon NF-κB activation. The IL-17RA expression was increased in IL-1R2 knockout mice after I/R injury. We showed in vivo and in vitro that IL-1R2 protects cardiomyocytes against apoptosis upon I/R injury by downregulating the IL-17RA/STAT1 signaling. Furthermore, IL-1R2 suppresses ROS production, leading to alleviation of cardiomyocytes damage after I/R injury.

Immune response plays a critical role in the healing of infarct tissue after myocardial I/R injury. However, emerging studies indicated that excessive inflammation induces cardiomyocyte injury. Cardiomyocytes go through a contraction-relaxation cycle to pump blood around the body [28], which is essential for life and makes it important to maintain cardiomyocytes' contractility. Recent studies have found that cardiomyocyte expressed cytokine receptors, such as Toll-like receptor 4 (TLR4) and glycoprotein 130 (gp130), which participated in cardiomyocyte apoptosis during I/R injury [29–31]. However, little was previously known about the IL-1R2 expression in cardiomyocyte. We demonstrated here that cardiac I/R injury significantly induced IL-1R2 expression in cardiomyocyte both in vivo and in vitro. In contrast, sIL-1R2 level was increased in the circulation of AMI patients who have undergone interventional therapy, which was relevant with poorer cardiac function. Similarly, Hilde found that sIL-1R2 level was independently relevant with parameters of LV adverse remodeling following ST-Elevation Myocardial infarction (STEMI), which suggested that sIL-1R2 could be a promising cardiac remodeling marker [32]. Previous reports have shown that circulating IL-1R2 was mainly expressed by neutrophils and monocytes under inflammatory conditions [18, 33]. Here, we found that upon H/R stimulation, NRVM accumulated sIL-1R2 both in the cytoplasm and cell-cultured medium, which indicated that elevated sIL-1R2 level derived from damaged cardiomyocyte after I/R injury. Besides neutrophils and monocytes, we reported for the first time that injured cardiomyocytes also contributed to producing IL-1R2 during the pathogenesis of I/R injury. What is more, we also observed that global knockout of IL-1R2 worsens cardiac dysfunction and increased cardiomyocyte apoptosis during I/R injury. Given that inflammatory cells regulated cardiac function upon I/R injury, silencing IL-1R2 expression in neutrophils and monocytes may increase IL-1β expression and inflammatory cells infiltration into ischemic area. Recently, it is reported that fibroblast-specific IL-1R1 expression was an important contributor to cardiac remodeling after myocardial infarction [29]. However, it remains unclear whether IL-1R2 is expressed in fibroblast or its effects on cardiac function upon I/R injury.

As a decoy receptor for IL-1β, Il-1R2 functions as a negative mediator to inhibit IL-1β signaling transduction and therefore reduce inflammatory. In our in vivo study, we found that IL-1R2 knockout mice increases neutrophil and macrophage infiltration into the ischemic region. Previous studies in ulcerative colitis have demonstrated that blocking IL-1R2 upregulated the expression of pro-inflammatory chemokines induced by Il-1β, such as TNF-a, IL-6 and IFN-γ [34]. Although there was no report showing that IL-1R2 reduced monocytes infiltration into heart during myocardial I/R injury, which was also our important discovery, some studies have suggested that IL-1R2 plays a role in regulating the monocytes accumulation during myocardial I/R injury. Canakinumab, an IL-1β-blocking antibody, was shown to improve cardiac function and reduce inflammation in mice [35]. In a pilot feasibility study of a patient with acute myocardial infarction, similar effects were achieved with Canakinumab treatment, in which Canakinumab treatment significantly reduced inflammation and heart failure incidence [36]. Therefore, we conclude that IL-1R2 contributes to attenuate monocytes accumulation by inhibiting IL-1β signaling transduction during myocardial I/R injury.

Although previous studies of IL-17A were largely focused on monocytes, such as CD4^+^ Th17 cells, recent research revealed that IL-17A bridges adaptive and innate immunity, providing a new insight to investigate the role of IL-17A in I/R injury. Yu *et al* found that the expression of IL-17A was elevated after myocardial I/R injury. Administration with IL-17A induced cardiomyocyte apoptosis, CXC chemokine-mediated neutrophil migration in vivo [37]. Even though IL-17RA expression was found to be induced in heart upon I/R injury [38], the mechanism is still unclear. In the current study, we found that IL-17RA expression was induced by IL-1β treatment and augmented with IL-1R2 knockdown in cardiomyocyte. Besides, by inhibiting the IL-17RA expression, IL-1R2 overexpression also reduced cardiomyocyte apoptosis, which was induced by the IL-17A. Studies about IL-1β showed IL-1β treatment on mice worsen cardiomyocyte damage upon I/R injury [39, 40]. However, the IL-1β/IL-17 signaling in cardiomyocyte was still unknown. As a decoy receptor for IL-1β, IL-1R2 participates in attenuating the IL-1β signaling transduction, which further induces IL-17RA expression and triggers cardiomyocyte apoptosis. Here we showed for the first time that by inhibiting IL-17RA expression, cardiomyocyte IL-1R2 plays an important role in reducing apoptosis in I/R injury.

It is known that IL-17A promoted both of STAT1 and STAT3 phosphorylation [41]. STAT1 is a transcription factor that is known to modulate cell apoptosis signaling. In myocardial I/R injury, STAT1 was activated in apoptotic cardiomyocytes and enhanced apoptosis by upregulating Bax expression. Some studies showed that pro-inflammatory cytokines, such as IL-1β and TNF, induced macrophages STAT1 phosphorylation [42]. Knockout of STAT1 protects heart against myocardial I/R injury [10]. Besides phosphorylated STAT3, we unexpectedly found that the phosphorylated STAT1 level was increased in IL-1R2 knockout mice after I/R injury in the present study. Consistent with this, the expression of p-STAT1 and Bax were also significantly induced in NRVM treated with IL-17A and IL-1β but reduced in IL-1R2 overexpressed NRVM in vitro. Collectively, these results indicate that IL-1R2 protects cardiomyocyte by downregulating IL-17A/IL-17RA/p-STAT1 signaling. Existing evidence showed that ROS induced by H_2_O_2_ enhanced STAT1 phosphorylation, which subsequently caused mitochondria dysfunction [43]. Additionally, STAT inhibition significantly reduced ROS production. In current study, we found that ROS production was suppressed in IL-1R2 overexpressed cardiomyocyte after I/R injury. These findings indicate that IL-1R2 reduces ROS production in cardiomyocyte by downregulating IL-17RA/STAT1 signaling. Interestingly, previous reports have suggested that the STAT-ROS cycle is essential for causing cardiomyocyte damage with I/R injury [44, 45]. Taken together, IL-1R2 protects cardiomyocytes from damage by prohibiting ROS production during myocardial I/R injury.

## Conclusions

In conclusion, we have revealed that myocardial I/R stress-induced cardiomyocyte IL-1R2 expression, which suppresses IL-17RA induction during myocardial I/R injury. Downregulating of IL-17RA attenuates IL-17A-induced cardiomyocyte apoptosis upon I/R injury. Thus, IL-1R2 overexpression in cardiomyocyte represents a novel target in myocardial I/R injury and paves a new way for further research to study cytokine receptor in cardiomyocyte.

## Materials and methods

### Human plasma isolation

The collection of blood plasma samples from AMI patients and healthy controls were conducted with approval from the National Health Commission of the People’s Republic of China. Blood was collected in 5 mL quantities using an EDTA tube and kept at 4 °C for 24 h. The upper yellowish portion was isolated by centrifugation at 3000 rpm for 5 min and then collected to store at −80 °C until used. All of the acute myocardial infarction patients were treated with statins to lower blood lipids. Besides, antihypertensive drugs, such as betaloc and ACEI, will used in patients with high blood pressure.

### ELISA assay

For the patient serum ELISA assay, Human IL-1R2 Quantikine ELISA kit (R&D systems) was purchased and the IL-1R2 levels were tested according to the manufacturer’s recommendations. For the NRVM-culture medium ELISA assay, the Rat IL-1R2 ELISA kit was purchased from Elabscience. For the mouse serum ELISA assay, the mouse IL-1R2 ELISA kit (Protentech) and mouse IL-1β ELISA kit (Abcam) were purchased and these tests were performed according to the manufacturer’s recommendations.

### Cardiac I/R surgery and TTC staining

All animal studies were conducted with approval from the Animal Welfare Ethics Committee of Run Run Shaw Hospital affiliated to Zhejiang University. Male C57BL/6 mice aged 8–10 weeks were subjected to cardiac I/R surgery as previously described [22, 46]. Briefly, animals were anesthetized with 0.3% pentobarbital sodium and placed on a warming pad to maintain body temperature. After inserting a tracheal cannula with a 19 G stump needle, mice were ventilated using a mouse ventilator (Shanghai Alcott Biotech Co., Ltd., Shanghai, China). Next, an oblique incision was made from the left sternal border, and the fourth intercostal muscle was incised to expose the left anterior descending coronary artery. A ligature was performed ~2 mm below the left auricle tip using a 6-0 silk thread. After 45 min of coronary occlusion, the ligation was released for tissue reperfusion. For 2,3,5-triphenyltetrazolium (TTC) staining, the animals were sacrificed 24 h after reperfusion and injected with 2% Evans blue directly into the aorta root to stain the non-ischemic zone. Subsequently, the heart was extracted, sliced, and then incubated in 2% TTC solution at 37 °C for 30 min. The left ventricle was divided into the ischemic, border, and remote regions according to the TTC staining patterns. For the western blots and qRT-PCR assays, the border region from the left ventricle was excised and placed in a thermos flask filled with liquid nitrogen. After tissue collection, they were transferred to store at −80 °C refrigerator until use. The ratio of 260/280 between 1.8–2.0 and the ratio of 260/230 between 2.0–2.2 were considered to be pure RNA.

### IL-1R2 knockout mice interbreeding

IL-1R2 knockout mice described previously were obtained from Cyagen Biosciences (Santa Clara, CA, USA). IL-1R2^+/-^ mice were interbred to generate IL-1R2^+/+^ and IL-1R2^−/−^ mice. IL-1R2^+/+^ mice were used as negative controls. The mice were housed in a temperature-controlled environment with 12-hour light/dark cycles.

### Generation and in vivo administration of AAV9

All animal studies were conducted with approval from the Animal Care and Use Committee of the Zhejiang University School of Medicine. To overexpress IL-1R2 in cardiomyocytes, a recombinant AAV9 expressing IL-1R2 (mm-IL-1R2-GCAGAGAGUUCAAAUCUGAdTdT) was purchased from Shanghai HanHeng Co (Shanghai, China), along with AAV9-GFP. The titers were estimated at ~1 × 1012 encapsidated genomes/mL.

Male C57BL/6 J mice aged 5–7 weeks were anesthetized with 0.3% pentobarbital sodium (25 mL kg-1) and ventilated through intubation. After making an incision in the fifth intercostal space to expose the heart, 40 µL of AAV9 expressing IL-1R2 or GFP was directly injected into four different sites in the expected ischemic region of the left ventricle. After injection, the thoracic cavity was sutured with a 4-0 silk thread. The hearts of all the injected animals were harvested after 3 weeks to examine IL-1R2 expression by western blotting or immunofluorescent staining.

### Isolation of NRVMs

The ventricles of (1–2)-day-old Sprague–Dawley rats were extracted, minced into small pieces, and then incubated in enzyme solution containing 0.114% collagenase II (Biosharp, China) and 0.036% trypsin (Solarbio, China) at 37 °C with gentle agitation according to previous reports [47, 48]. After sufficient digestion and clearance of fibroblasts by differential centrifugation, cardiomyocytes were collected and then plated in a cell-culture dish with high glucose DMEM medium containing 10% FBS and 1% penicillin and streptomycin. Besides, 100 μM bromodeoxyuridine was added to the culture medium to prevent the growth of contaminating fibroblasts. After that, NRVMs were cultured in DMEM without serum for 24 h followed by experiments.

### Stimulated I/R in NRVM

To stimulate I/R in NRVMs, we prepared fresh ischemia Esumi buffer (4 mM HEPES, 117 mM NaCl, 12 mM KCl, 0.9 mM CaCl2, 0.49 mM MgCl2, 20 mM sodium lactate, 5.6 mM 2-deoxyglucose, pH 6.2), as previously reported [22]. After washing the NRVMs with PBS twice to remove the culture medium, cells were incubated at 37 °C in a hypoxia chamber (STEMCELL, US) with a gas mixture of 94.8% N2/5% CO2/0.02% O2. After 3 h of hypoxia, the Esumi buffer was replaced with the standard culture medium and the cells were incubated at 37 °C, 5%CO2 for 3 to 12 h.

### siRNA and plasmid transfection

Small interfering RNAs (siRNAs) were purchased from Sigma-Aldrich (NM_053953, St. Louis, MO, USA) and transfected into NRVMs using Lipofectamine RNAiMAX (Invitrogen). The IL-1R2 overexpression plasmid was purchased from Shanghai Genechem Co., Ltd (NM_004633; Shanghai, China) and transfected into NRVM by using Lipofectamine 3000 Transfection Kit (Invitrogen) as previously described. Briefly, 1 × 106 NRVMs were plated in a 6-well plate and cultured with high glucose DMEM medium containing 10% FBS. After 24 h, the culture medium was changed with 600 μL Opti-MEM (Life Technologies Corporation) mixed with 0.8 μL plasmid-IL-1R2 (50 μM) and 6 μL lipofectamine 3000 for 6 h. Next, the Opti-MEM solution was replaced with high glucose DMEM medium and NRVMs were cultured for 48 h for thorough transfection.

### Western blotting

The mouse hearts or NRVMs were lysed using RIPA lysis buffer (Solarbio, R0020) with a protease-inhibitor cocktail (Beyotime, P1030). After normalizing the total protein concentrations via the BCA analysis (Beyotime, P0010), the lysates were boiled in loading buffer for 10 min. Afterward, 20 µg lysate per sample was resolved in SDS-polyacrylamide gels (FD341; Hangzhou Fude Biological Technology Co., Ltd. Hangzhou, China), followed by transfer onto PVDF membranes for western blotting. After blocking with 5% skimmed milk for 1 h, the membranes were incubated with antibodies against IL-1R1 (1:1000, Santa Cruz, Biotechnology, Santa Clara, CA, USA), IL-1R2 (1:1000, Novus Biologicals, Littleton, CO, USA), p-NF-κB (1:1000, CST), JAK2 (1:1000, CST), p-JAK2 (1:1000, CST), STAT1 (1:1000, CST), p-STAT1 (1:1000, CST), STAT3 (1:1000, CST), p-STAT3 (1:1000, CST), Bcl-xL (1:1000, CST), Bax (1:1000, Proteintech, Rosemont, IL, USA), IL-17RA (1:1000, Abclonal, Wuhan, China), nitrotyrosine (1:1000, Sigma-Aldrich) and GAPDH (1:5000, CST) overnight at 4 °C. Afterward, the membranes were washed with TBST (2% Tween) and incubated with HRP-conjugated secondary antibodies for 1 h at room temperature. A chemiluminescence HRP substrate (Bio-Rad) and ChemiDoc Imaging System (Bio-Rad) were used to detect the protein bands.

### Immunofluorescence and TUNEL assays

Paraffin sections were prepared from heart tissues with myocardial I/R injury. After deparaffinization in xylenes and antigen retrieval, these sections were blocked with 5% BSA for 1 h at room temperature followed by incubation with antibodies against IL-1R2 (1:200, Novus Biologicals, Littleton, CO, USA) and cTnT (1:200, Abcam, Cambridge, UK) were performed to these sections at 4 °C overnight. For diminishing the interference of GFP expression, we treated the sections with 100% methanol for 10 min before blocking with 5% BSA solution. After washing with PBS, the samples were incubated with Alexa Fluor-594–conjugated goat anti-mouse antibodies and Alexa Fluor-488–conjugated anti-rabbit antibodies [1:400; MultiSciences (Lianke) Biotech Co., Ltd. Hangzhou, China] for 1 h at room temperature. Afterward, they were counterstained with DAPI and imaged using a Nikon A1 microscope (Tokyo, Japan). The TUNEL staining was performed according to the manufacturer’s protocol (Roche, Basel, Switzerland) and imaged by a Nikon A1 microscope. The numbers of TUNEL-positive cardiomyocyte nuclei were quantified with Image J software (NIH, Bethesda, MD, USA). Three separate fields of view were quantified within the border region of infarction from each section to average the positive myocytes.

### Echocardiography

The cardiac contractile functions of the control and experimental mice were assessed under anesthesia via echocardiography using a Vevo 1100 system (Visual Sonics, Toronto, Canada), as previously described [49]. M-mode recordings were made, and ventricular fractional shortening (FS%) and ejection fraction (EF%) were examined.

### RNA isolation and gene expression analysis

Total cardiac RNA was extracted using TRIzol (Invitrogen, Carlsbad, CA, USA) according to the instruction manual, and the RNA quality and quantity were assessed using a NanoDrop and Agilent 2100 bioanalyzer (Thermo Fisher Scientific, MA, USA). Total mRNA was converted into complementary DNA (cDNA) by PrimeScript RT reagent kit (Takara, Tokyo, Japan). Subsequently, quantitative PCR was performed by using a PCR mixture (11203ES08, Yeason, China) and a LightCycler 480 II system (Roche). Target mRNA levels were normalized to *gapdh* mRNA levels and reported as fold changes relative to the control group. The sequences of the primers were as follows in Supplemental Table 2.

### mRNA library construction and RNA-seq analysis of gene expression

Total RNA from heart tissues of IL-1R2^+/+^ and IL-1R2^−/−^ mice were extracted by Trizol reagent (Invitrogen, CA, USA) and analysis of Bioanalyzer 2100 and RNA 6000 Nano LabChip Kit (Agilent, CA, USA) with RIN number > 7.0. Following purification, the mRNA is fragmented into small pieces using divalent cations under elevated temperatures. Then the cleaved RNA fragments were reverse-transcribed to create the final cDNA library in accordance with the protocol for the mRNA-Seq sample preparation kit (Illumina, San Diego, USA).

For sequence and primary analysis, a cDNA library constructed by technology from the pooled RNA from heart samples of mice with myocardial I/R injury was sequenced run with Illumina sequence platform (LC-Bio Technology co.ltd., Hangzhou, China). A total of millon paired-end reads of bp length were generated by using the Illumina paired-end RNA-seq approach. The low-quality reads (1, reads containing sequencing adapters; 2, reads containing sequencing primer; 3, nucleotide with *q* quality score < 20) were removed. After a total of G bp of cleaned, paired-end reads were produced, the raw sequence data were submitted to the NCBI Short Read Archive with accession number.

For transcript abundance estimation and differentially expressed testing, all transcriptomes were merged to reconstruct a comprehensive transcriptome using Perl scripts. After the final transcriptome was generated, StringTie and Ballgown were used to estimate the expression levels of all transcripts. StringTie was used to perform expression level for mRNAs by calculating FPKM. The differentially expressed mRNAs and genes were selected with log2 (fold change) > 1 or log2 (fold change) < −1 and with statistical significance (*p*-value < 0.05) by R package—Ballgown. The false discovery rate (FDR) < 0.05 was employed.

### Flow cytometry

To quantify cell death, NRVM were harvested by 0.05% trypsin without EDTA and neutralized with fetal bovine serum. After washing in cold PBS twice, annexin V-FITC and PI staining solution (DOJINDO LABORATORIES) were added according to the manufacturer’s recommendations. Next, 300 µL detecting solution was added for suspension and the stained cells were analyzed by flow cytometry (BD FACSCalibur).

### Statistics

All data are expressed as mean ± SEM. The student’s *t*-test (2-tailed) and one-way ANOVA with Tukey’s test were used for statistical analysis in GraphPad Prism 8. *P* < 0.05 was considered statistically significant.

## Supplementary information

Supplemental legends

Supplemental table 1

Supplemental table 2

Supplemental Figure 1

Supplemental Figure 2

Supplemental Figure 3

Supplemental Figure 4

Supplemental Figure 5

## Data Availability

The datasets generated and/or analyzed during the current study are available from the corresponding author on reasonable request.
