# Supplementary material for: Cardiomyocyte IL-1R2 protects heart from ischemia/reperfusion injury by attenuating IL-17RA-mediated cardiomyocyte apoptosis
Source: Cell Death Dis. 2022 Jan 27;13(1):90. doi: 10.1038/s41419-022-04533-1 (PMC8795442; doi:10.1038/s41419-022-04533-1)
Supplement: Supplementary file 1 — Supplemental legends [file 41419_2022_4533_MOESM1_ESM.docx]

**Supplemental figure legends**

**Supplemental Figure 1 Protocol figures for in vivo and in vitro investigations.** (**A**) protocol of myocardial I/R injury; (**B**) protocol of NRVM stimulated with H/R treatment; (**C**) protocol of myocardial I/R injury in IL-1R2+/+ and IL-1R2-/- mice; (**D**) protocol of AAV9 injection followed by myocardial I/R injury in male mice.

**Supplemental Figure 2. Strategy for building IL-1R2 knockout mice and siRNA for knocking down of IL-1R2 expression in NRVM.** (**A**) LDH assay of control and NRVM with 3 h of hypoxia followed by 3, 6 and 12 h of reoxygenation. (**B-C**) Representative blots and quantification of the NF-κB inhibition effect by PDTC treatment. (**D**) Genotyping strategy for producing IL-1R2 knockout mice. (**E-F**) PCR screening and RT-PCR analysis of F1 pups for genotyping IL-1R2 knockout mice. (**G**) qRT-PCR for IL-1R2 in IL-1R2 knockout mice and control littermates. **P<0.0 and 1-way ANOVA for 3 groups or more.

**Supplemental Figure 3 Knockout of IL-1R2 induces RAGE and TNF pathway expression.** (**A**) Heatmap of IL-1R2+/+ and IL-1R2-/- mouse mRNAs clustering from heart tissue after I/R injury. (**B**) Relative FPKM levels of genes related to RAGE pathway in IL-1R2+/+ and IL-1R2-/- mice with myocardial I/R injury (n=3). The differentially expressed mRNAs and genes were selected with log2 (fold change) >1 or log2 (fold change) <-1 and with statistical significance (p value < 0.05). (**C**) Relative mRNA levels of genes related to TNF pathway in IL-1R2+/+ and IL-1R2-/- mice with myocardial I/R injury (n=3). (**D-E**) Representative images and quantification of the effect of IL-1R2 reducing the NRVM apoptosis induced by IL-17A. *P<0.05 and **P<0.01. 2-tailed unpaired Student’s t test for 2 groups or 1-way ANOVA for 3 groups or more.

**Supplemental Figure 4. IL-1β induces IL-17RA expression in cardiomyocyte via P38 MAPK signaling, which was abrogated by cardiomyocyte IL-1R2 overexpression.** (**A**) Representative western blots of IL-17RA expression in NRVM with IL-1β treatment at different timepoints. (**B**) Representative western blots of IL-17RA expression in NRVMs with SB 203580 (P38 MAPK inhibitor) or SP 600125 (JNK inhibitor) treatment followed by IL-1β treatment for 24 hours. (**C**) Representative western blots of knocking down IL-1R2 in NRVM with siRNA-1, siRNA-2 and siRNA-3 treatment. (**D**) Representative western blots of IL-1R2 expression in NRVMs with IL-1R2-overexpression plasmid transfection. (**E**) Protein level of sIL-1R2 from cell cultured supernatant of NRVMs with or without IL-1R2 overexpression (n=3). (**F-G**) Representative images and quantification of TUNEL and cTnT staining with or without overexpression of IL-1R2 followed by IL-1β and IL-17A treatment. Scale bar: 100 µm. *P<0.05 and **P<0.01. 2-tailed unpaired Student’s t test for 2 groups or 1-way ANOVA for 3 groups or more.

**Supplemental Figure 5 IL-1R2 protects heart from I/R injury via suppression of ROS production.** (**A**) Representative images of TUNEL staining on the heart sections from the Sham (n=3), GFP (n=5) and IL-1R2–overexpressing (n=5) mice subjected to myocardial I/R surgery. (**B**) Serum level of IL-1R2 in sham (n=5), GFP and IL-1R2 overexpression mice (n=5) subjected to myocardial I/R surgery. (**C-D**) Representative images and blots of nitrotyrosine expression in the hearts of the GFP and IL-1R2–overexpressing mice subjected to myocardial I/R injury. Scale bar: 100 µm. (**E-F**) Western blot analysis of the hearts from GFP and IL-1R2–overexpressing mice subjected to myocardial I/R injury for nitrotyrosine expression. (**G**) Quantification of IL-1R2 overexpression in cardiomyocyte by AAV9 injection (n=3). (**H**) Quantification of the effect of siRNA silencing the IL-1R2 expression in NRVM (n=3). (**I**) Lactate dehydrogenase (LDH) assay of NRVMs with or without IL-1R2 silencing by siRNA transfection followed by hypoxia/reoxygenation treatment. *P<0.05 and **P<0.01. 2-tailed unpaired Student’s t test for 2 groups or 1-way ANOVA for 3 groups or more.

**Supplemental table 1 Clinical characteristics of healthy controls and AMI patients enrolled**

**Supplemental table 2 Relative primer sequences**
