# Supplementary material for: Cardiomyocyte IL-1R2 protects heart from ischemia/reperfusion injury by attenuating IL-17RA-mediated cardiomyocyte apoptosis
Source: Cell Death Dis. 2022 Jan 27;13(1):90. doi: 10.1038/s41419-022-04533-1 (PMC8795442; doi:10.1038/s41419-022-04533-1)
Supplement: Supplementary file 2 — Supplemental table 1 [file 41419_2022_4533_MOESM2_ESM.docx]

**Table 1 Clinical characteristics of healthy controls and AMI patients enrolled**

|  | **Control** | **AMI** |
| --- | --- | --- |
| Age (years) mean (SD) | 53.10±7.68 | 61.00±10.44 |
| Sex |  |  |
| Women (%) | 40 | 40 |
| Man (%) | 60 | 60 |
| Body mass index (kg/m^2^) | 21.77±2.81 | 24.05±4.02 |
| Smoking % | 30 | 50 |
| Blood pressure |  |  |
| Systolic BP. mmHg | 118.7±11.67 | 120.42±12.14 |
| Diastolic BP, mmHg | 68.7±10.65 | 72.33+13.87 |
| Blood lipid |  |  |
| Triglyceride, mmol/L | 1.3±0.95 | 1.35±0.5 |
| Total cholesterol, mmol/L | 4.71±1.19 | 3.88±1.06 |
| High-density lipoprotein, mmol/L | 1.12±0.3 | 1.02±0.14 |
| Low Density Lipoprotein, mmol/L | 2.23±0.91 | 2.18±0.72 |
| Medical history |  |  |
| Hypertension (yes), % | 30 | 33 |
| Diabetes mellitus (yes), % | 30 | 33 |
