# Supplementary material for: Cardiomyocyte IL-1R2 protects heart from ischemia/reperfusion injury by attenuating IL-17RA-mediated cardiomyocyte apoptosis
Source: Cell Death Dis. 2022 Jan 27;13(1):90. doi: 10.1038/s41419-022-04533-1 (PMC8795442; doi:10.1038/s41419-022-04533-1)
Supplement: Supplementary file 3 — Supplemental table 2 [file 41419_2022_4533_MOESM3_ESM.docx]

**Table 2 Relative primer sequences**

| **Gene** | **Forward (5’-3’)** | **Reverse (5’-3’)** |
| --- | --- | --- |
| IL-1R2 (mice) | GATCCAGTCACAAGGGAGGA | CCAGGAGAACGTGGAAGAGA |
| IL-1R1 (mice) | GAGTTACCCGAGGTCCAGTGG | GAGGGCTCAGGATAACAGG |
| GAPDH (mice) | TCACCACCATGGAGAAGGC | GCTAAGCAGTTGGTGGTGCA |
| IL-1R2 (Rat) | CATTCAGACACCTCCAGCAGTTC | ACCCAGAGCGTATCATCCTTCAC |
| IL-1R1 (Rat) | GTTTTTGGAACACCCTTCAGCC | ACGAAGCAGATGAACGGATAGC |
| GAPDH (Rat) | CAACTCCCTCAAGATTGTCAGCAA | GGCATGGACTGTGGTCATGA |
