# Supplementary figures and images for: Cardiomyocyte IL-1R2 protects heart from ischemia/reperfusion injury by attenuating IL-17RA-mediated cardiomyocyte apoptosis
Source: Cell Death Dis. 2022 Jan 27;13(1):90. doi: 10.1038/s41419-022-04533-1 (PMC8795442; doi:10.1038/s41419-022-04533-1)

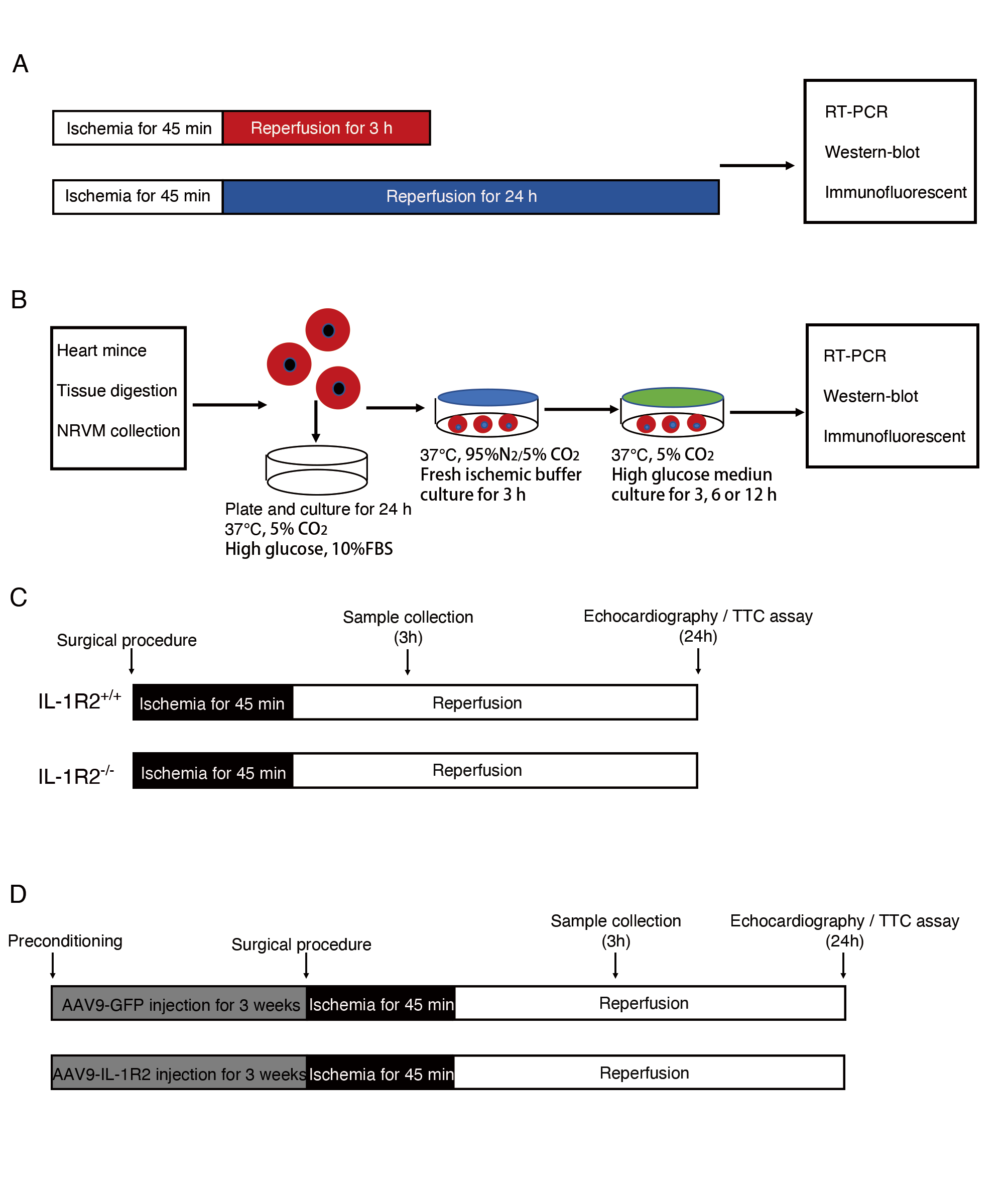

Supplement: Supplementary file 4 — Supplemental Figure 1 [file 41419_2022_4533_MOESM4_ESM.png]

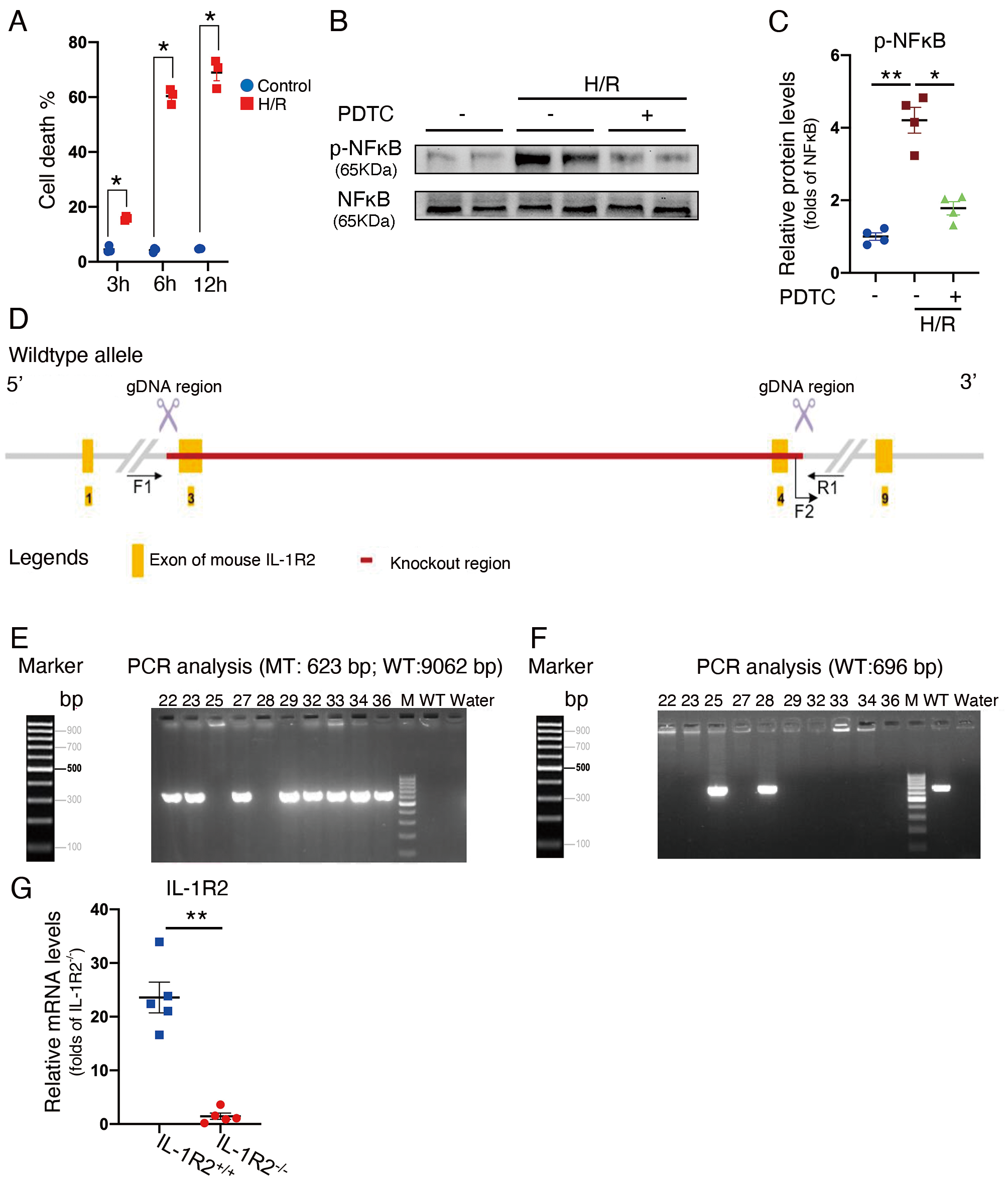

Supplement: Supplementary file 5 — Supplemental Figure 2 [file 41419_2022_4533_MOESM5_ESM.png]

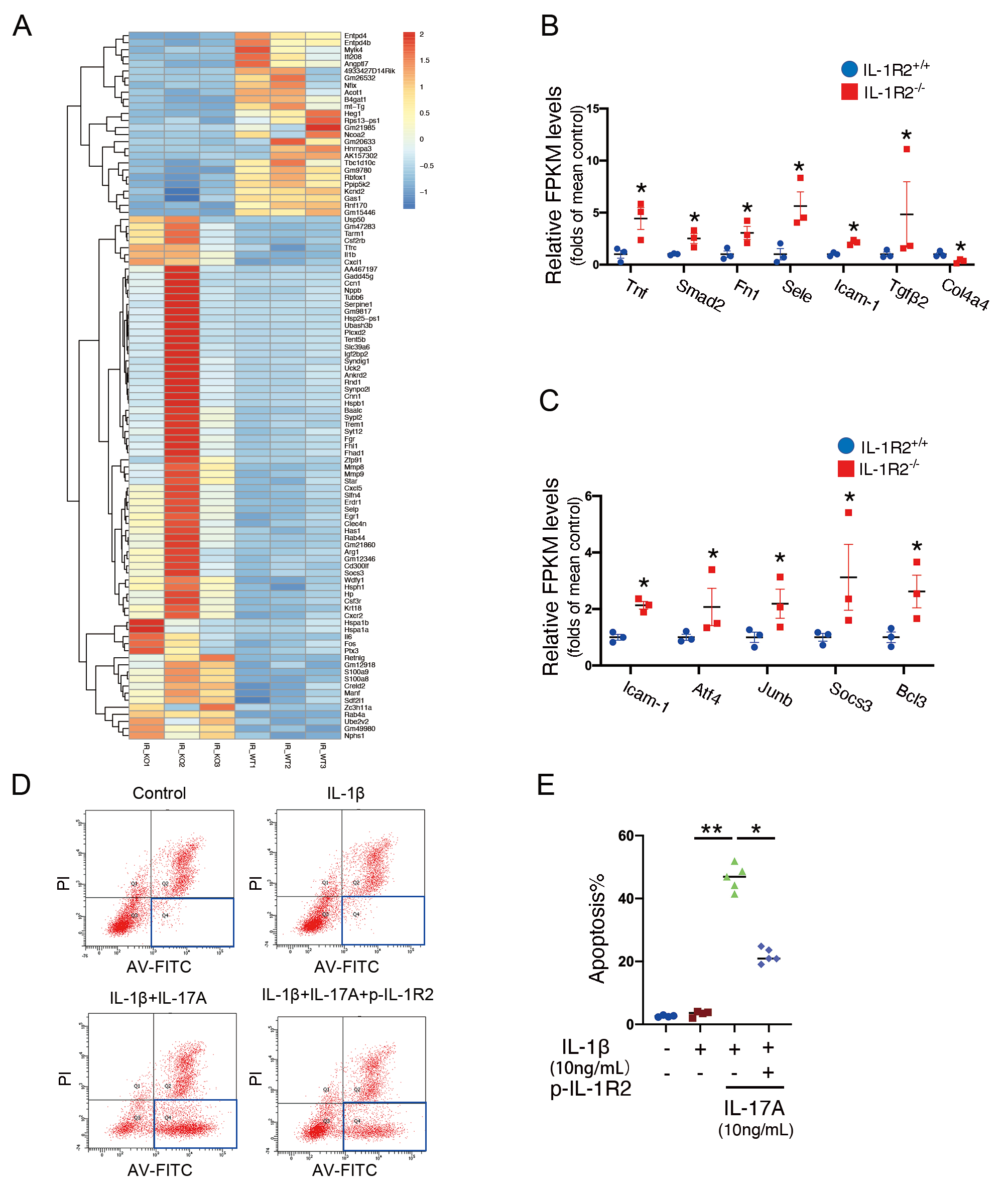

Supplement: Supplementary file 6 — Supplemental Figure 3 [file 41419_2022_4533_MOESM6_ESM.png]

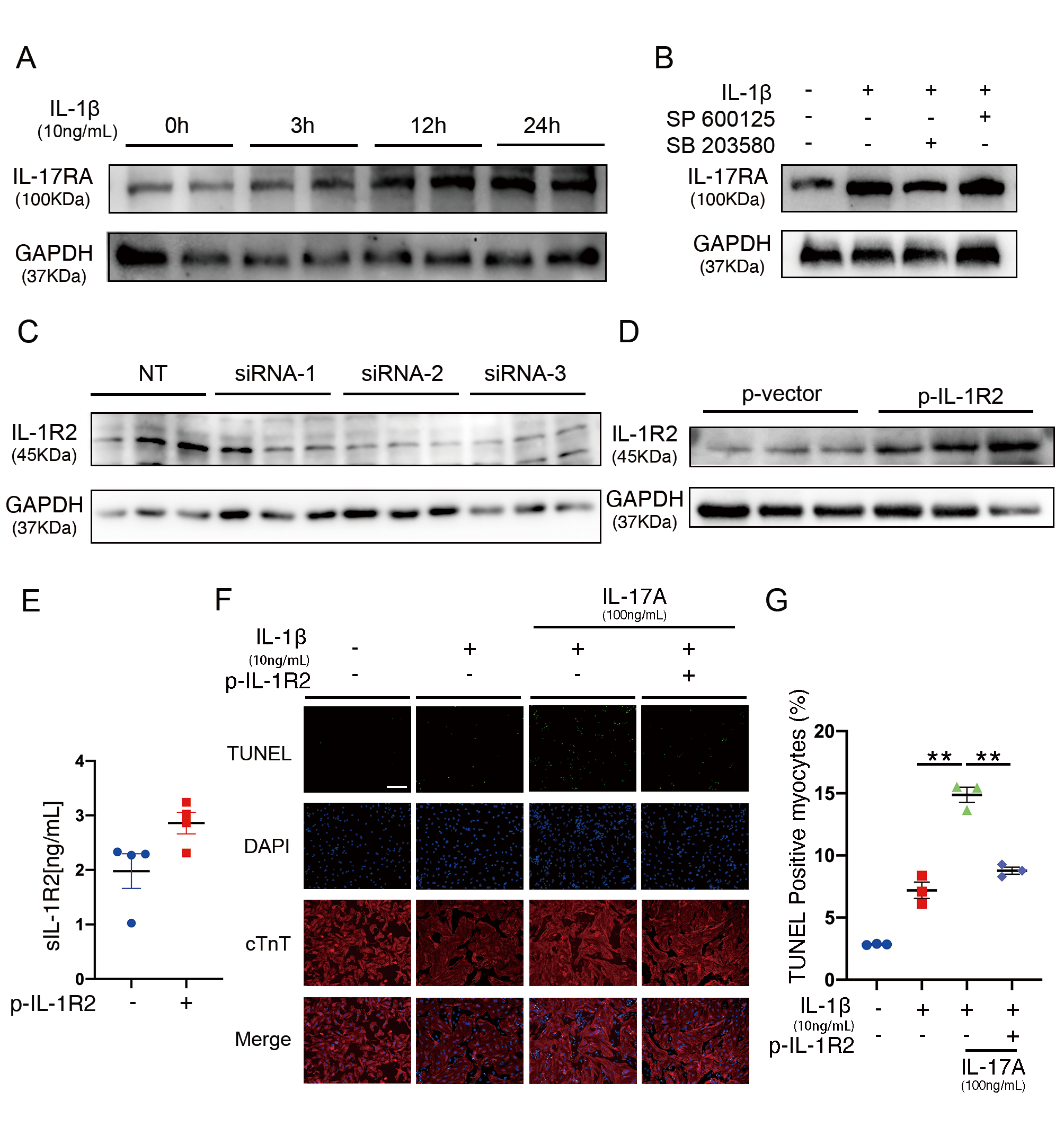

Supplement: Supplementary file 7 — Supplemental Figure 4 [file 41419_2022_4533_MOESM7_ESM.png]

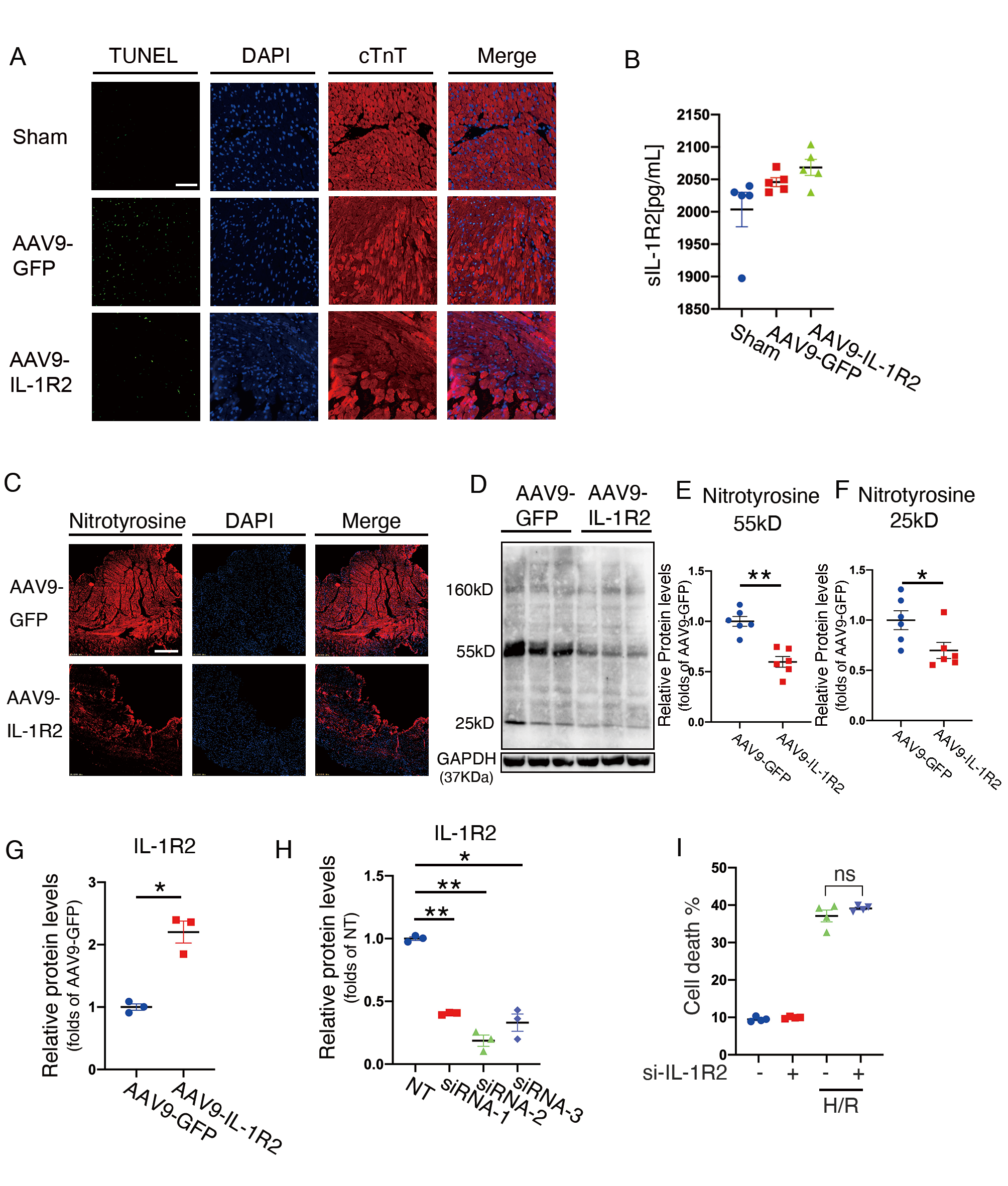

Supplement: Supplementary file 8 — Supplemental Figure 5 [file 41419_2022_4533_MOESM8_ESM.png]
